# Editorial: Advances in technology-assisted rehabilitation

**DOI:** 10.3389/fresc.2024.1465671

**Published:** 2024-08-06

**Authors:** Andreas Kannenberg, Rüdiger Rupp, Shane R. Wurdeman, Laurent Frossard

**Affiliations:** ^1^ Department of Clinical Research & Services, Otto Bock Healthcare LP, Austin, TX, United States; ^2^ Spinal Cord Injury Center, Heidelberg University Hospital, University of Heidelberg, Heidelberg, Germany; ^3^ Hanger Institute of Clinical Research & Education, Hanger, Inc., Austin, TX, United States; ^4^ Griffith Centre of Biomedical and Rehabilitation Engineering, Menzies Health Institute, Griffith University, Gold Coast, QLD, Australia

**Keywords:** rehabilitation, technology-assisted care delivery, prosthetics, orthotics, robotics, wearable sensors

**Editorial on the Research Topic**
Advances in technology-assisted rehabilitation

## 1 Context

The 2011 World Report on Disability of the World Health Organization (WHO) estimated that more than a billion people worldwide—about 15% of the 2010 global population—experience some form of disability (1). In 2019, a study estimated that more than 2.4 billion people globally are affected by conditions that could benefit from rehabilitation (2). These numbers have only been growing due to population ageing and increase in the prevalence of non-communicable diseases (1, 3). Chronic diseases are estimated to account for 66.5% of all years lived with disability (4). Altogether, these projections are likely to increase the socio-economic burden of diseases requiring rehabilitation, including costs on healthcare systems already under tremendous financial pressure.

Individual disability results from the interaction between impairments of the overall physical and mental state and particular health condition of body parts or systems as well as personal and environmental factors (e.g., negative attitudes towards people with a disability, lack of motivation in self-care management, lack of access to transportation and public buildings, limited social support) (5). The environment of a person has a huge impact on the experience and extent of a disability. Inaccessible environments may create barriers to the full and effective participation of persons with disabilities in society on an equal basis with others (e.g., distance between home and closest point of care). Progress on improving social participation can be made by addressing these barriers and facilitating these persons with disabilities in their lives (e.g., role of carer, peer support) (1, 3).

Rehabilitation addresses the impact of an impaired health condition on a person's everyday life by optimizing their function and reducing the experience of disability. Rehabilitation ensures people with an impairment of a body structure and function (or mental functioning) can remain as independent as possible and participate in meaningful life roles through education, work, and recreational activities. Global demographics and health trends, such as population ageing, medical workforce shortages, rising prevalence of non-communicable diseases, as well as continued consequences of conflict, injury and developmental conditions are placing increasing demands on the health care systems. The need for safer, more efficient and cost-effective rehabilitation interventions (e.g., devices, programs, therapies) is rapidly growing, yet in many parts of the world this need is largely unmet (1).

Altogether, improving effectiveness and efficiency of technology-assisted rehabilitation may contribute to address the challenges associated with the increasing needs for high-quality rehabilitation under conditions of limited human and financial resources (6–10). For that reason, substantial attention and resources have been directed at rehabilitative and assistive technologies recently. As an example, the WHO started the Global Cooperation on Assistive Technology in 2014 (8) and published the first-ever Global Guide on Assistive Technology in 2021 (10).

One type of technology for rehabilitation of motor functions can support professionals, such as physical and occupational therapists, in providing physically demanding trainings to patients allowing them to reach their maximum potential to live an independent life again. For example, such technologies can support patients to actively participate in restorative trainings with multiple repetitions of movement tasks, in documenting treatment progress, and potentially also in the ability to oversee and direct the treatment of more than just one patient at a time (11–15).

Another type of rehabilitation technologies, such as prostheses, orthoses, wearable sensors or functional electrical stimulation garments can directly assist and support people with motor impairments to achieve their daily lives activities goals. For example, these technologies can help restore motor functions such as grasping and walking by compensating for permanently lost anatomical structures, such as after an amputation, and/or diminished or lost function due to injury or central nervous or neuromuscular disorders (16).

Unfortunately, the successful translation of technological innovations into rehabilitation settings and patients’ lives often remains limited (17). It is estimated that only about 10% of patients receive the assistive devices they need. This inequity is even worse in low- and middle-income countries (8, 17). Interestingly, though health care experiences growing staffing shortages, especially in the developed countries, there still appears to be resistance among healthcare providers to adopt assistive technologies (11, 18). This might be partially due to the combination of an abundance of informative technical publications (e.g., proof of concept, early outcomes with prototype) as well as the scarcity of high-quality research on clinical outcomes (e.g., randomized clinical trial to assess safety and efficacy) and the absence of realistic health economic evaluations [e.g., cost-utility analyses (19)]. Therefore, it appears prudent to research the effectiveness of assistive and rehabilitation technologies and to create a dedicated venue for the publication of such research.

## 2 Scope of the research topic

The research topic of technology-assisted rehabilitation was intentionally defined broadly to address a wide spectrum of topics starting from technology development including perspectives on determination of patient needs and demands, continuing with clinical studies with prototypes and commercially available devices, clinical research to address regulatory and/or reimbursement requirements, and ending with health-economic research with assistive technology to support in- and out-patient rehabilitation and/or temporary or long-term everyday home use.

## 3 Contributions

### 3.1 Outline of contributions

In total, 19 manuscripts were submitted for review and 16 papers from 89 authors (30% female) from 41 institutions across 6 countries were accepted for publication in this research topic. It presents 12 original research articles, 2 literature reviews, and one policy and practice review and perspective each.

### 3.2 Ethical perspectives of technology development

Gavette et al. provide a perspective on the ethical considerations surrounding the development and translation of prosthetic technologies into clinical practice that have received little attention to in the past. Based on current literature, they present perspectives from their multidisciplinary views as prosthetists, researchers in prosthetics on wearable technologies for rehabilitation, machine learning, artificial intelligence, and ethics of advanced technologies. The authors discuss ethical considerations for current advances in prosthetic technology, as well as topics for future research, that may inform product and policy decisions and positively influence the lives of patients.

### 3.3 Policy and practice review

Jones et al. present a summary of findings and recommendations of two multi-stakeholder workshops to address research gaps and requirements defined by the National Health Service (NHS) England to adopt coverage of multi-grip myoelectric prosthetic hands. The workshops involved people from a broad range of stakeholder groups and discussed design requirements for policy-driven research studies and research questions identified in the policy review. The consented recommendations include the need for qualitative and quantitative research evidence, use of goal-based outcome measures, conduct of longitudinal studies, and addressing of the complexity of national and international policy-driven research, such as clinical resource capacity and participant involvement.

### 3.4 Original research articles—upper limb prosthetics

Simon et al. performed a study on the further advancement of pattern recognition systems for the control of upper limb prostheses. The study enrolled six individuals with no upper limb absence and four persons with transradial amputation who controlled a virtual prosthesis with the current standard 8-channel or 16-channel EMG pattern recognition. Participants had significant improvements in control when using 16 compared to 8 EMG channels including decreased classification error and decreased completion time. Scores of the Assessment for Capacity of Myoelectric Control (ACMC) increased by more than three times the minimal detectable change from the 8 to the 16-channel condition.

Maas et al. conducted a randomized controlled study on technology-assisted motor learning to optimize training of myoelectric control of upper limb prosthesis. Thirty-six participants with no motor impairments were randomly assigned to either a task-specific serious game training group, a non-task-specific serious game training group, or a control group using a computer mouse. Differences between groups over test sessions lacked a systematic structure and were not significant. The authors concluded that transfer effects from game training to actual prosthesis use did not take place in the non-disabled study participants. However, an important finding was that significant individual differences were found which not just means that motor learning is different for each person but that these individual differences should be considered in future studies and their translation to rehabilitation practice.

### 3.5 Original research articles—lower limb prosthetics

Monaghan et al. performed a retrospective review of health records of 174 individuals with unilateral transtibial limb loss who received care at Walter Reed National Military Medical Center (Bethesda, Maryland, USA) between 2001 and 2019 to analyze prescription patterns for the first prosthetic foot after amputation. They identified patient-specific characteristics, such as sex, time between injury and initial prescription, time from amputation to initial prescription, and amputation etiology that influenced initial ankle-foot prosthesis prescription. Using these factors as predictors, they were able to correctly classify 72% of all first prosthetic feet prescribed proving a systematic prescription pattern over almost two decades.

Tacca et al. pursued a new approach to evaluating the mechanical properties of prosthetic feet. They characterized stiffness values and hysteresis of 33 stiffness categories and sizes of a commercially available prosthetic foot with and without a shoe. They found that foot size had a significant impact on axial and torsional stiffness values and hysteresis within the same manufacturer-defined stiffness category, and that use of a shoe had also a significant impact on stiffness. Their results suggest manufacturers should adjust the design of prosthetic feet in each stiffness category to ensure mechanical properties are consistent across different sizes and highlight the need to consider the effects of shoes.

Klute et al. conducted a clinical study with a novel, torsionally active ankle-foot prototype prosthesis (TAP) that can generate transverse plane rotation trajectories proportional to sagittal plane ankle angles corresponding at varying coupling ratios. Eleven individuals with unilateral transtibial amputation walked in a straight line and in both directions around a circle with the TAP set at randomized coupling ratios. The general pattern of results suggested a quadratic relationship between the peak transverse plane moment and coupling ratio with a minimum at the 6:1 coupling ratio. The coupling ratio did not appear to adversely affect propulsion or body support. Subjects indicated they found all coupling ratios to be comfortable.

Herrin et al. report a new approach to optimize the individual tuning of a tethered, research-grade powered prosthetic foot using eight different metrics of gait quality in seven individuals with unilateral transtibial amputation. Differences between the tuned and untuned conditions were reflected in several parameters, with improvements seen in all of them during use of the tuned prosthesis. All these metrics relate to the timing of force generation during walking, which is information not directly accessible to a prosthetist in everyday clinic. This work indicates that real-time biomechanical data provided to the prosthetist may improve future clinical tuning procedures for powered prostheses.

Klenow et al. performed a study with an updated microprocessor-controlled prosthetic knee (MPK—Genium™, Otto Bock, Duderstadt, Germany) with newly developed parameter presets for individuals with bilateral transfemoral amputation. A convenience sample of 17 unilateral and 9 bilateral MPK users was recruited for the study that assessed a battery of performance-based and patient-reported outcome measures. Stumble frequency was significantly reduced by 85% with the updated Genium MPK. The bilateral group reported significant 50% and 57% greater relative improvements in patient-reported ease and safety, respectively, of completing activities of daily living (ADL) compared to the unilateral group.

Krout et al. report early research efforts to manage low-level weight bearing to help maintain perfusion and improve proprioception and residual limb tissue health in transtibial prosthesis users. The goal of the project was to develop a sensor to measure distal weight bearing and to evaluate socket design variables that affect weight bearing. Participants accepted weight-bearing levels ranging from 1.1% to 6.4% of body weight. Two of the three participants preferred distal weight bearing over non-presence. The next steps will be to determine target weight bearing levels and ranges, and to simplify the sensor and socket adjustment mechanism for clinical use.

Dickinson et al. conducted a retrospective chart review of socket rectifications in 134 randomly selected prosthetic users using 163 CAD/CAM transtibial sockets to assist future socket design choices. Limb and socket scans were compared to determine individual rectification of patella tendon bearing (PTB) and total surface bearing (TSB) socket designs, and associations between different rectification sizes were assessed using a variety of methods. Differences in design features were apparent between sockets, notably for paratibial carves, gross volume reduction, and distal end elongation. Design patterns were consistent with expert clinician practice. This study demonstrates how we might learn from design records to support education and enhance evidence-based socket design.

Leister et al. performed a study comparing the daily step count measured in 79 participants with transtibial amputation with the affordable but unvalidated FitBit Inspire 3 and the research-grade, validated activPAL. The study results show that the FitBit Inspire 3 counted 1,094 ± 1,423 more steps per day than the activPAL. However, a high correlation between the results of both monitors was found. Because of the significant mean differences, the activPAL and FitBit Inspire 3 are not interchangeable for estimating physical activity in persons with transtibial amputation. However, due to the high correlation of results, the consistent application of each of the devices results in similar classification rankings based on step counts.

### 3.6 Original research article and systematic literature review—bone-anchored prosthetics

Gladish et al. report the characterization of mechanical loads distal to the percutaneous part of the osseointegrated implant for fitting bone-anchored prostheses in four male individuals, two with unilateral and two with bilateral transfemoral amputation. Tri-directional forces and moments were wirelessly recorded through a sensor during six functional tests. Peak mechanical loads were largest during non-steady state components of the functional tests (e.g., side-stepping, standing up from the ground). Relative to walking, peak forces during functional tests were 110% to 181%, and peak moments 108% to 211% larger. The results allow for a more comprehensive understanding of the mechanical loads applied to bone-anchored implants, which is critical to maximize implant survivability and long-term outcomes.

Rehani et al. presents a systematic review of the literature on outcomes, complications, patient experiences, and cost-effectiveness of transfemoral bone-anchored prostheses, in which thirty-eight studies were included. The most common study design was the single-arm pre-/post-intervention trial. The clinical efficacy of bone-anchored prostheses was evident in selected populations. Overall, patients reported increased health-related quality of life, mobility, and prosthesis usage. The most common complication was a superficial or soft-tissue infection, while more serious complications were rare. The evidence from literature indicates that bone-anchored prostheses are cost-effective for those individuals who face significant challenges in using socket-suspension systems.

### 3.7 Original research article and literature review—lower limb orthotics

Hovorka et al. performed a study that investigated the neuromuscular output during the early adaptation period to constraint of ankle joint motion. Electromyography (EMG) of calf muscles was used to monitor muscle activation output in non-disabled individuals between constrained and unconstrained ankle motion using an ankle-foot orthosis (AFO) combined with footwear. Results support an emergent theory that when ankle joint motion is constrained during walking, skeletal muscle activation of uniarticular muscles acting on the constrained ankle joint is altered. Thus, clinicians need to be aware of this adaptive response period particularly in users that do not have a neuromotor deficit.

LeCursi et al. report on a study that aimed at proposing an explicit methodology for the adjustment of multi-function articulated AFOs in the clinical setting. Multi-function articulated AFOs offer features that permit more comprehensive and reversible adjustments of AFO ankle alignment and resistance to ankle motion. However, no standard method exists for the application and optimization of these therapeutic devices. Published evidence supporting most decision points of the algorithm is presented, two hypothetical case examples are given to illustrate the application of the method to the optimization of articulated AFOs, and gaps in evidence in this respect were identified.

## 4 From product idea to clinical standard

The papers published in this research topic are intended to motivate researchers and clinicians to engage in product development, clinical research, and compilation of peer-reviewed publications that further advance innovations in technology-assisted rehabilitation. Generation of evidence, preferably through registered clinical trials of high methodological quality are a necessity and prerequisite for widespread clinical adoption and acceptance as standard of care by healthcare payers. Robust, independent evidence for long-term effects and benefits of innovations will also be necessary to overcome the so called “decline effect”. This describes the phenomenon of initially strong results of new treatment options in early studies conducted by the developers that are later contrasted with more realistic results of independent, bigger studies with longer follow-ups (20). This will be critical to convince public and private healthcare funding bodies to support a particular innovation, particularly with the emergence of value-based reimbursement models (e.g., hospital, physicians, workmańs compensation, and insurance) (21–23).
